# Extending chemical perturbations of the ubiquitin fitness landscape in a classroom setting reveals new constraints on sequence tolerance

**DOI:** 10.1242/bio.036103

**Published:** 2018-07-15

**Authors:** David Mavor, Kyle A. Barlow, Daniel Asarnow, Yuliya Birman, Derek Britain, Weilin Chen, Evan M. Green, Lillian R. Kenner, Bruk Mensa, Leanna S. Morinishi, Charlotte A. Nelson, Erin M. Poss, Pooja Suresh, Ruilin Tian, Taylor Arhar, Beatrice E. Ary, David P. Bauer, Ian D. Bergman, Rachel M. Brunetti, Cynthia M. Chio, Shizhong A. Dai, Miles S. Dickinson, Susanna K. Elledge, Cole V. M. Helsell, Nathan L. Hendel, Emily Kang, Nadja Kern, Matvei S. Khoroshkin, Lisa L. Kirkemo, Greyson R. Lewis, Kevin Lou, Wesley M. Marin, Alison M. Maxwell, Peter F. McTigue, Douglas Myers-Turnbull, Tamas L. Nagy, Andrew M. Natale, Keely Oltion, Sergei Pourmal, Gabriel K. Reder, Nicholas J. Rettko, Peter J. Rohweder, Daniel M. C Schwarz, Sophia K. Tan, Paul V. Thomas, Ryan W. Tibble, Jason P. Town, Mary K. Tsai, Fatima S. Ugur, Douglas R. Wassarman, Alexander M. Wolff, Taia S. Wu, Derek Bogdanoff, Jennifer Li, Kurt S. Thorn, Shane O'Conchúir, Danielle L. Swaney, Eric D. Chow, Hiten D. Madhani, Sy Redding, Daniel N. Bolon, Tanja Kortemme, Joseph L. DeRisi, Martin Kampmann, James S. Fraser

**Affiliations:** 1 Biophysics Graduate Group, University of California, San Francisco 94158, USA; 2 Bioinformatics Graduate Group, University of California, San Francisco 94158, USA; 3 Chemistry and Chemical Biology Graduate Program, University of California, San Francisco 94158, USA; 4 Department of Biochemistry and Biophysics, University of California, San Francisco 94158, USA; 5 Department of Chemistry Undergraduate Program, University of California, Davis 95616, USA; 6 Department of Bioengineering and Therapeutic Sciences, California Institute for Quantitative Biology (QBI), San Francisco 94158, USA; 7 Department of Cellular and Molecular Pharmacology, California Institute for Quantitative Biology (QBI), San Francisco 94158, USA; 8 Department of Biochemistry and Molecular Pharmacology, University of Massachusetts Medical School, Worcester 01655, USA; 9 Institute for Neurodegenerative Diseases, University of California, San Francisco 94158, USA

**Keywords:** Deep mutational scanning, Evolution, Ubiquitin

## Abstract

Although the primary protein sequence of ubiquitin (Ub) is extremely stable over evolutionary time, it is highly tolerant to mutation during selection experiments performed in the laboratory. We have proposed that this discrepancy results from the difference between fitness under laboratory culture conditions and the selective pressures in changing environments over evolutionary timescales. Building on our previous work (Mavor et al., 2016), we used deep mutational scanning to determine how twelve new chemicals (3-Amino-1,2,4-triazole, 5-fluorocytosine, Amphotericin B, CaCl_2_, Cerulenin, Cobalt Acetate, Menadione, Nickel Chloride, p-Fluorophenylalanine, Rapamycin, Tamoxifen, and Tunicamycin) reveal novel mutational sensitivities of ubiquitin residues. Collectively, our experiments have identified eight new sensitizing conditions for Lys63 and uncovered a sensitizing condition for every position in Ub except Ser57 and Gln62. By determining the ubiquitin fitness landscape under different chemical constraints, our work helps to resolve the inconsistencies between deep mutational scanning experiments and sequence conservation over evolutionary timescales.

## Abstract (precis)

**Summary:** We organized a project-based course that used deep mutational scanning of ubiquitin to resolve the inconsistencies between tolerance to mutations in laboratory conditions and sequence conservation over evolutionary timescales.

## INTRODUCTION

The increased capabilities of deep sequencing technologies have transformed our ability to interrogate pooled libraries of variants under selection or screening conditions (Fowler and Fields, 2014). In particular, protein sequence-structure-function studies are benefiting from the ability to comprehensively survey the functional effects of all possible single point mutants in experiments that have come to be called ‘deep mutational scans’ (Araya and Fowler, 2011; McLaughlin et al., 2012). Deep mutational scanning experiments are revealing new dimensions of protein stability (Araya et al., 2012), substrate specificity (Shah et al., 2018; Wrenbeck et al., 2017) and regulation (Bandaru et al., 2017). These experiments also provide insight into the evolutionary significance of the spectrum of mutational effects on fitness. For example, recent studies have also used deep mutational scanning to probe how mutations are tolerated in different sequence backgrounds, reflecting the local sequence space tolerated over long evolutionary timescales (Starr et al., 2017, 2018). These experiments rely on the connection between the character of the laboratory selection (or screen) and the pressures experienced by populations of organisms in the natural environment. Indeed, comparing phylogenetic analysis of naturally occurring variation between homologs with deep mutational scanning data of a single protein can reveal sites that are experiencing different selective pressures in nature versus the laboratory (Hilton et al., 2017). Across many studies, a general trend has emerged with the expected general correlation between sites that are poorly conserved in evolution tolerating more substitutions more readily in deep mutational scanning experiments and with highly conserved sites being less tolerant to substitutions.

One interesting contrast to the general trends between evolutionary sequence conservation and deep mutational scanning tolerance to substitution is the protein ubiquitin (Ub), an essential eukaryotic protein that acts as post-translational modification to mediate the degradation of ∼80% of the proteome (Yau and Rape, 2016) and is also one of the first proteins subjected to a yeast-based deep mutational scanning experiment (Roscoe et al., 2013). The amino acid sequence of ubiquitin has been strikingly stable throughout evolutionary time: between yeast and humans, there are only three amino acid changes (96% sequence identity) (Finley et al., 2012). However, deep mutational scanning experiments in yeast have revealed that Ub is surprisingly robust to sequence changes, with 19 positions freely mutating to almost any other amino acid without a loss of fitness (Roscoe et al., 2013). Some of this pattern could be rationalized structurally: a sensitive structural surface is the known interface for many binding partners, whereas the positions that are tolerant to mutation make few contacts with structurally characterized binding partners (Roscoe et al., 2013). However, the outstanding disconnect between strong constraints during natural evolution and tolerance during laboratory selection remained unaddressed by the original study.

To interrogate the dichotomy between the strong sequence conservation and the mutational robustness of Ub, we initially hypothesized that sensitivities to mutations at new positions could be revealed by growing yeast under different selective pressures. To test this idea we previously determined, in a classroom setting, the fitness landscape of ubiquitin in four different chemical perturbations [DTT, caffeine, hydroxyurea (HU), and MG132] (Mavor et al., 2016). We showed that three of the perturbations (DTT, caffeine and HU) sensitize a shared set of positions to mutation, including several positions that were not sensitive to mutation under the standard growth conditions employed previously. Conversely, we showed that the proteasome inhibitor MG132 increases the mutational robustness of the ubiquitin sequence landscape. Inhibiting the proteasome reduces protein turnover through the same pathway as mutations in ubiquitin, leading to an alleviating interaction between MG132 and many of the mutant alleles. A major conclusion from this study was that the fitness defects, relevant for rationalizing evolutionary patterns, were buffered and undetectable in standard laboratory growth, but that these defects could be unmasked by simple chemical stresses. However, 12 of the 19 residues, the residues classified as ‘tolerant’ [almost all mutations at that position have near wild-type (WT) fitness] in standard growth conditions, were still tolerant under all chemical stresses.

To identify potential environmental perturbations that could help to rationalize the constraint on the ‘tolerant’ residues over evolutionary time, we again involved the first-year graduate students in UCSF's iPQB and CCB programs to determine the fitness landscape of ubiquitin in distinct environments. We chose twelve new chemical perturbations [3-Amino-1,2,4-triazole (3-AT), 5-fluorocytosine (5-FC), Amphotericin B (AmpB), CaCl_2_, Cerulenin, Cobalt Acetate (Cobalt), Menadione, Nickel Chloride (Nickel), p-Fluorophenylalanine (p-FP), Rapamycin, Tamoxifen, and Tunicamycin], which were expected to impose a wide range of stresses upon the cell, including osmotic shock, protein folding stress and DNA damage. By using these additional stresses, we can now identify laboratory conditions that place strong constraints on the sequence preferences of all but two residues in ubiquitin. Our results represent an important next step towards how deep mutational scanning can be used to explain the evolutionary constraints on sequence conservation patterns.

## RESULTS

### Distinct chemical treatments can sensitize or increase robustness of Ub to mutation

**Fig. 1.**
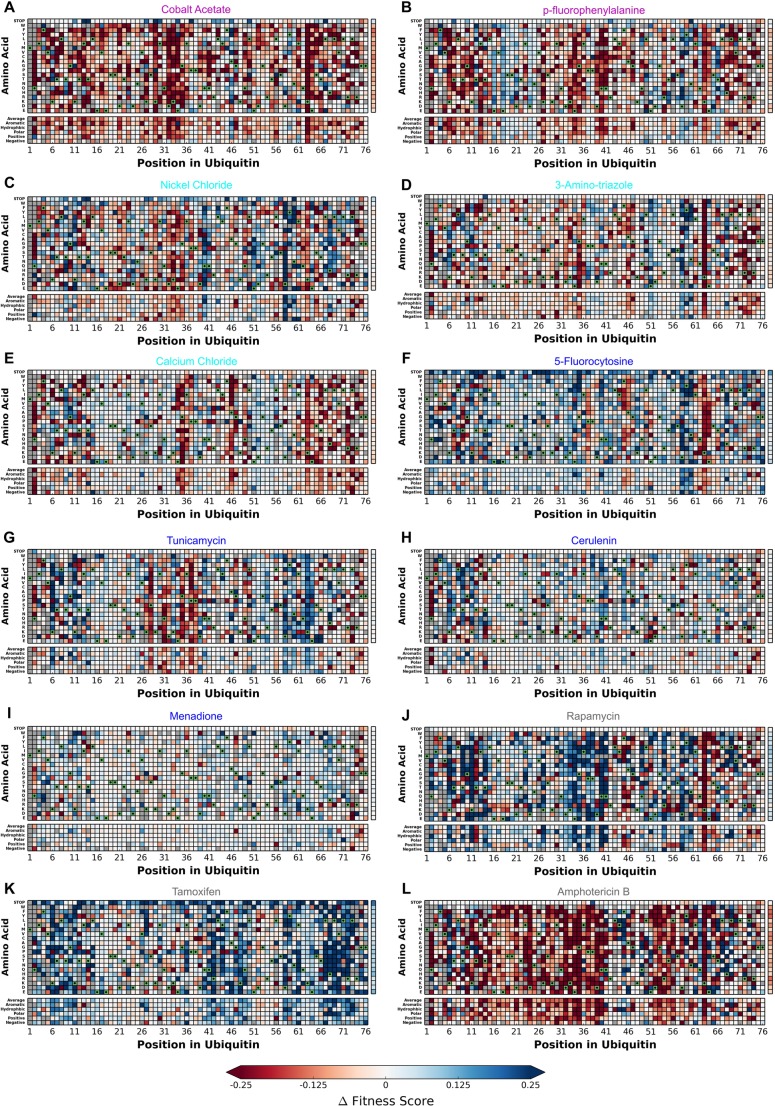
**The difference in fitness between DMSO and a perturbation for each Ub allele.** Chemical names are colored based on the hierarchical clustering presented in Fig. 1: (A) Cobalt, (B) p-FP, (C) Nickel, (D) 3-AT, (E) CaCl_2_, (F) 5-FC, (G) Tunicamycin, (H) Cerulenin, (I) Menadione, (J) Rapamycin, (K) Tamoxifen, (L) AmpB. Difference in fitness is represented from 0.25 (Blue) to −0.25 (Red) with white representing no change from DMSO. Wild-type amino acids are shown in green and mutations without fitness values (due to lack of barcode or competition sequencing reads) are shown in grey.

As in our previous work, we performed deep mutational scanning experiments of a barcoded Ub library in the presence of distinct chemical perturbations at concentrations that inhibited the growth of a strain expressing wild-type ubiquitin by 25% (Mavor et al., 2016). To quantify the effect of the chemical on growth of specific mutations, we subtracted the fitness values of our control (DMSO) dataset and generated difference fitness maps (Fig. 1). The most obvious global trends are the increased sensitization of many residues when treated with AmpB (Fig. 1L) and increased robustness of many residues when treated with Tamoxifen (Fig. 1K). In contrast, treatment with Menadione leads to a very similar pattern of fitness effects to control treatments (Fig. 1I).

**Fig. 2.**
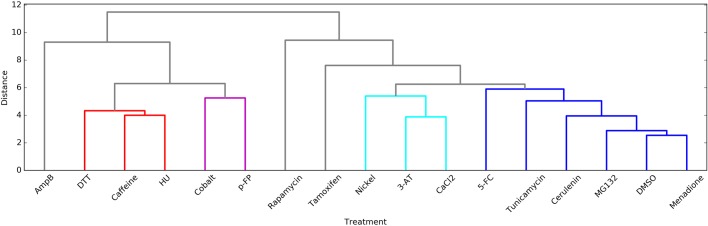
**Hierarchical clustering of the fitnesses reveals four distinct clusters.** Treatment with Cobalt and p-FP (magenta) cluster together and close to the previously described ‘sensitizing treatments’ (Mavor et al., 2016), DTT, Caffeine and HU (red). Treatment with Menadione, Cerulenin, Tunicamycin and 5-FC cluster with DMSO and the previously described ‘alleviating treatment’ MG132 (blue).

To place these results in context of our previous chemical treatments (Mavor et al., 2016), we employed hierarchical clustering based on the pattern of fitness effects across each chemical treatment (Fig. 2). The treatments with Cobalt and p-FP form a new cluster near the previously described ‘sensitizing’ treatments DTT, Caffeine and Hydroxyurea (Fig. 2). These treatments share a sensitizing effect at positions near hydrophobic patch residues (8, 44, 70) and the C-terminus (Fig. 1). In contrast, treatment with Cerulenin, Menadione, Tunicamycin, and 5-FC clustered near to treatment with DMSO and MG132 (Fig. 2). These treatments are mild with many positions displaying mildly increased robustness to mutation and a few distinct mutations for each condition displaying stronger sensitization (Fig. 1).

Treatment with Nickel, 3-AT, or CaCl_2_ form a second novel cluster (Fig. 2). These treatments share a dominant pattern of sensitizing positions 35, 46 and 63 coupled with increased robustness at position 58 (Fig. 1). Three treatments demonstrated more idiosyncratic responses (AmpB, Rapamycin, and Tamoxifen) and do not cluster with other treatments (Fig. 1). These treatments induced either extreme sensitization of residues (AmpB), extreme increased robustness of residues (Tamoxifen), or positional dependent, but strong, sensitization and robustness (Rapamycin) (Fig. 2). Collectively, these results demonstrate that many of our new perturbations tap into similar constraints on tolerated sequence space for ubiquitin as our previous study, but also that many of our new perturbations likely unmask distinct constraints.

### Deep mutational scanning in different chemical environments reveals constraints on most residues

**Fig. 3.**
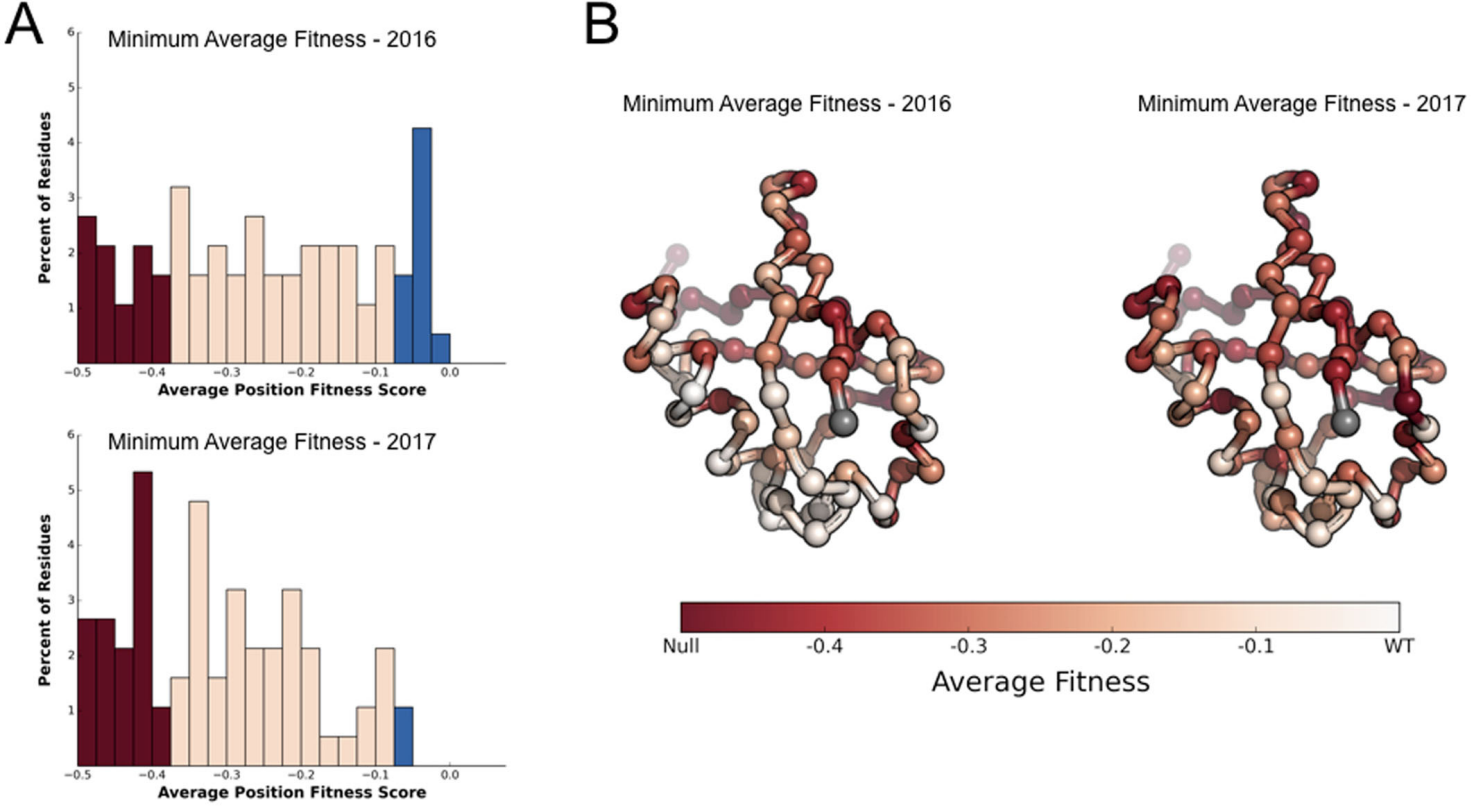
**New perturbations reveal constraints on all but two Ub positions.** (A) The minimum average fitness of each position was calculated in: (top) DMSO, Caffeine, DTT, HU and MG132 and (bottom) in all conditions. Minimum average fitness was determined by calculating the average fitness of each position in each condition and taking the minimum value. Positions were binned into tolerant (≥−0.075 - Blue), intermediate (<−0.075 to >−0.35 - Pink) and sensitive (≤−0.35 - Red) and the distributions plotted. Calculating the minimum average fitness reveals how the new perturbations reveal additional constraints on the Ub fitness landscape. (B) Minimum average fitness score in: (left) DMSO, Caffeine, DTT, HU, and MG132 and (right) in all conditions mapped onto the Ub structure. C-alpha atoms are shown in spheres and the residues are colored according to average fitness. Met1 is colored grey. Treatment with Nickel, 3-AT and CaCl_2_ cluster together (cyan) and close to the ‘alleviating treatment’ cluster. Treatment with AmpB, Rapamycin and Tamoxifen appear as outliers in this clustering (grey). The clustering was performed using euclidean distance between the vectors and used Ward's method to join the clusters. Clusters are colored based on the treatments being within 6 distance of each other.

To examine whether the new perturbations could help explain the high sequence conservation of Ub, we calculated the average fitness at each position for each condition. At each position, we used the fitness value from the condition with the lowest average fitness value and classified these minimum values based on the previous schemes (Mavor et al., 2016; Roscoe et al., 2013) as either sensitive (≤−0.35), intermediate (−0.35 to −0.075) and tolerant (≥−0.075) (Fig. 3). Previously we showed that twelve positions in Ub remained tolerant under the four different chemical stresses (Mavor et al., 2016). By expanding the number of perturbations, we now find that all but two positions, Ser57 and Gln62, are sensitive or intermediate in at least one condition. Although there is significant overlap in response of the Ub fitness landscape to these different perturbations, this result suggests that further exploration of chemical space might unmask constraints on the two residues for which a sensitizing condition has not yet been identified.

### Principal component analysis of deep mutational scanning data across chemical perturbations

**Fig. 4.**
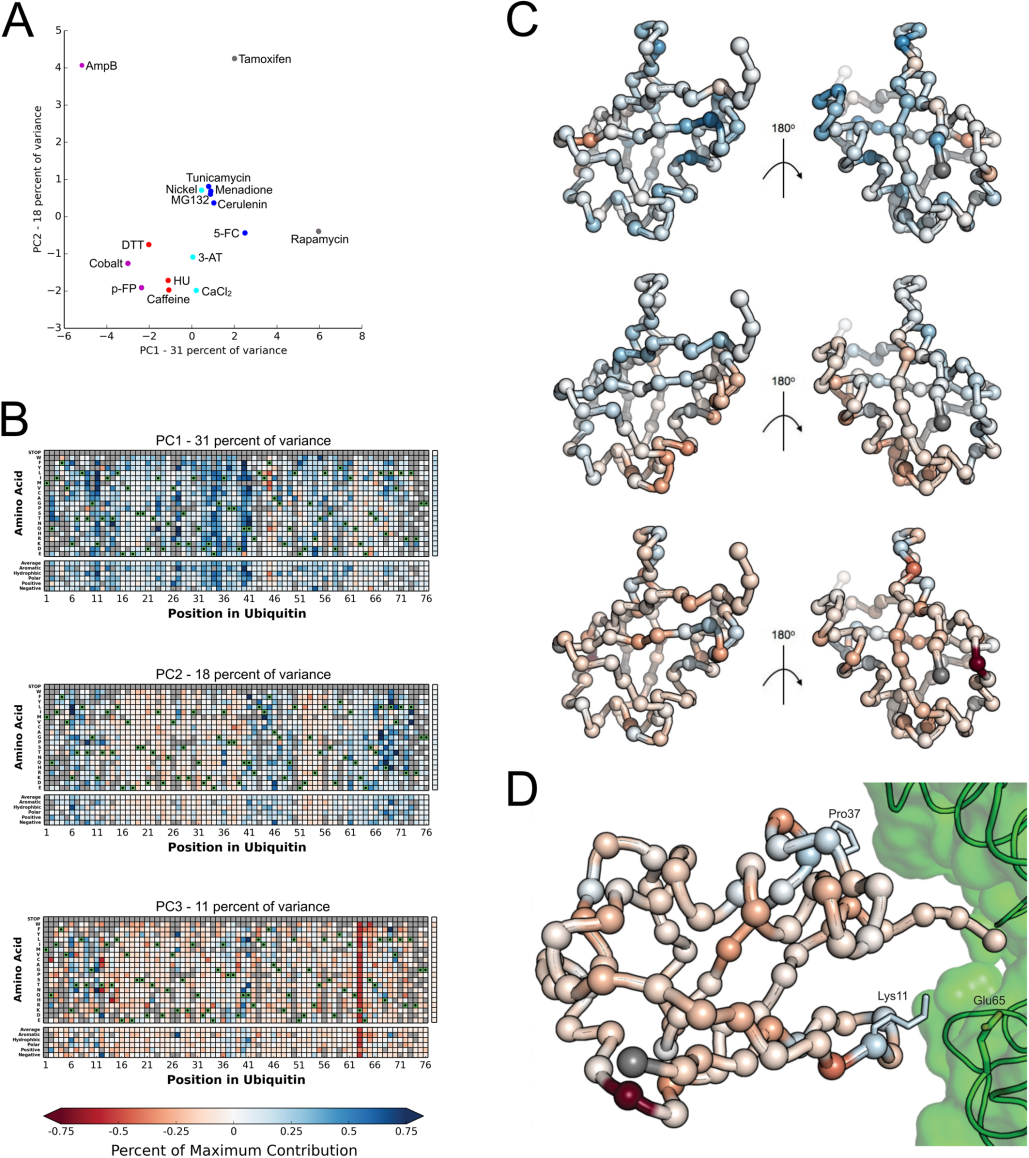
**Principal component analysis reveals specific signals related to K63 incorporation.** (A) The first two principal components reveal a sensitizing cluster with negative values in each PC and an alleviating cluster in the center of the plot. Treatment with 3-AT, 5-FC or CaCl_2_ appear between clusters with positive values in PC1 and negative values in PC2. The points are colored based on the hierarchical clustering shown in Fig. 1. (B) The contribution of each mutation to each principal component was visualized as a heat map. The percentage of the maximum contribution to that principal component is represented from 75% (Blue) to −75% (Red). PC1 is related to the general sensitivity of Ub mutants to perturbation. Regions with large positive contributions to PC1 correspond to the regions with increased mutational sensitivity in the sensitizing treatments. Strikingly this is coupled to negative contributions to PC1 for some mutations at the core residue Phe45 (top). PC2 differentiates ‘Sensitive Face’ residues (positive contributions) from ‘Tolerant Face’ residues (Negative Contributions) (middle). PC3 reveals mutations that are correlated with sensitization of Lys63 (bottom). (C) The average contribution of each mutation to a PC at each position was plotted from 75% (Blue) to −75% (Red) on the Ub monomer structure for PC1 (A), PC2 (B), and PC3 (C). (D) Rosetta ΔΔG calculations revealed that mutations that strongly destabilize the donor Ub pose on the MMS/Ubc13 (2GMI) heterodimer are localized to Lys11 and Pro37 (shown in sticks). In the case of Lys11, all mutations other than that to Arg destabilize the interface suggesting a salt bridge between Lys11 and Glu65 of Ubc13 (shown in green sticks) ubiquitin residues are colored by the contribution to PC3 as in panel C.

To explore whether correlated patterns of fitness values across treatment conditions could provide mechanistic insight into the Ub sequence-structure-function relationship, we performed principal component analysis on the difference fitness data (Fig. 4). We focused our analysis on the first three principal components, which collectively explain 60 percent of the variance (Fig. S1). Projecting the treatments onto the first two principal components reveals two main clusters in this space that parallel many aspects of the hierarchical clustering performed earlier (Fig. 2). Consistent with the hierarchical clustering, treatment with AmpB, Rapamycin, or Tamoxifen appear as outliers (Fig. 4A).

Next, we mapped the contribution of each mutation to each of the first three principal components (PCs) by primary sequence (Fig. 4B) and three dimensional structure (Fig. 4C). PC1 is dominated by mild positive contributions for most mutations, with the strongest positive signals appearing at residues 11, 27, 40 and 41. Interestingly, the strongest negative contributions appear at Phe45, a large core residue that may be involved in long-range correlated motions that are important for recognition of Ub by interacting proteins (Fenwick et al., 2011). PC2 is most similar to the initial description of the fitness landscape in rich media (Roscoe et al., 2013): both are dominated by the contrast between positive contributions from the tolerant face of Ub and the negative contributions from the sensitive face, which includes the ‘hydrophobic patch’ that forms the interface for most interacting proteins.

The most notable feature of PC3 is the response to mutation at Lys63, a key poly-Ub linkage site (Fig. 3B). In yeast, Lys63-linked poly-Ub is an important regulator of the DNA damage response and efficient intracellular cargo trafficking (Erpapazoglou et al., 2014). Since the other mutations with strong signals exposed by PC3 were not near K63 structurally (Fig. 3C), we investigated whether the pattern could be rationalized by examining structural complexes important for K63 linkages. We used the molecular modeling program Rosetta (Alford et al., 2017) to calculate the expected change in free energy of each mutation in various complexes involved in Lys63 linked poly-Ub assembly: the closed and open forms of Lys63 linked di-Ub (PDB ID: 2N2K (Liu et al., 2015) and 3H7P (Weeks et al., 2009)) and the donor and acceptor ubiquitin poses on the MMS/Ubc13 complex (PDB ID: 2GMI (Eddins et al., 2006)) (Fig. 3D; Fig. S2). Of these, only the MMS/Ubc13 donor Ub complex revealed any pattern correlated with PC3 (Fig. S2). Several positions predicted to destabilize this interface (including mutations at Lys11 and Pro37) have positive contributions to PC3 (Fig. 3D). This result suggests that conditions that are sensitized when K63-linked poly-Ub chains are compromised might have an increased relative fitness when positions that destabilize the donor ubiquitin pose are mutated. Consistent with this hypothesis is a mutational pattern observed for Lys11, which participates in a salt bridge with Glu65 of Ubc13. The Lys11Arg mutation, which is predicted to maintain the salt bridge and is the only mutation at position 11 that is predicted to stabilize the interface, has a negative contribution to PC3. Collectively, these results suggest that deep mutational scans from multiple chemical perturbations might reveal correlated responses that are difficult to uncover when analyzing only a single condition.

## DISCUSSION

No single perturbation in the laboratory can easily replicate the diverse pressures that natively constrain protein evolution. However, in the case of ubiquitin, we can now rationalize the extreme sequence conservation of Ub after examining the fitness landscape under a large variety of conditions that included redox stress, osmotic stress, protein folding stress, DNA damage, ER stress, and anti-fungals. Notable exceptions are residues Ser57 and Gln62, which are not sensitive to mutation under any condition yet tested.

Of the newly revealed sensitivities, perhaps the most interesting is the sensitization of Lys63. This sensitivity to mutation dominated the third principal component (PC3) and is present in eight conditions. Traditionally, Lys63-linked poly-Ub is thought to participate in the response to DNA damage, where Lys63-linked poly-Ub chains form on PCNA to induce error-free postreplication repair (Zhang et al., 2011), and in endocytosis, where efficient endocytosis in cargo sorting to the vacuole requires Lys63-linked poly-Ub chains (Erpapazoglou et al., 2014). More recent studies (Kwon and Ciechanover, 2017; Silva et al., 2015) have shown that Lys63 chains are involved in the yeast response to oxidative stress and autophagy in metazoans, suggesting that the role of Lys63-linked poly-Ub chains may be more extensive than its previously recognized role in DNA damage and endocytosis.

In contrast, we previously observed an increase in mutational robustness at Lys63 in DTT treatment, a reducing agent that interferes with ER protein folding. Interestingly, we also observed increased robustness under Tunicamycin treatment, a compound that interferes with ER protein folding via a distinct mechanism (Chawla et al., 2011). This result suggests an epistatic interaction between Lys63 signaling and the unfolded protein response, which may complement the suggested role of Lys11 under high (30 mM) DTT treatment (Xu et al., 2009). The Lys11Arg mutant is specifically sensitized in Tunicamycin suggesting that the origin of this effect may be structural, rather than due to a requirement for Lys11-linked poly-Ub.

In addition to the increased robustness at Lys63, Tunicamycin treatment leads to a unique increase in mutational robustness at several other positions, including Lys6, Lys11, and Lys33. These results address a major challenge in Ub biology: understanding the biological role of distinct poly-Ub species. While the mutational tolerance pattern at Lys6 and Lys11 appear to be due to disrupting a salt bridge, the increased robustness at Lys33 suggests a connection between Tunicamycin and Lys33 linked poly-Ub. We observed, further, but less conclusive, Lysine-specific effects for Lys27, Lys29, and Lys33 under treatment with AmpB, Cobalt, or Nickel.

Finally, these experiments continue to highlight the success of project-based courses. Building on our first effort (Mavor et al., 2016), we improved on our model: over the course of 6 weeks, first year graduate students in UCSF's CCB and iPQB programs generated and analyzed these data using their own computational pipelines. We believe that yeast-based deep mutational scanning experiments present ideal systems for such project-based courses due to the low cost and wide range of stress responses accessible by readily purchasable and common chemicals. It is our hope that other graduate programs can offer similar project based classes in the future and we have made our regents, code, and course material available to further that goal.

## MATERIALS AND METHODS

### Additional material is available

PUBS website (www.fraserlab.com/pubs).

GitHub (https://github.com/fraser-lab/PUBS).

Raw Sequencing reads are available via SRA (SRA Accession Number:SRP070953).

### Updated methods from Mavor et al. (2016)

For each compound, we determined the chemical concentrations that inhibited SUB328 (WT Ub) growth by 25% (3-Amino-1,2,4-triazole, 50 mM; 5-fluorocytosine, 1.25 μg/ml; Amphotericin B, 400 nM; CaCl_2_, 500 mM; Cerulenin, 4.5 μM; Cobalt Acetate, 600 μM; Menadione, 500 μM; Nickel Chloride, 400 μM; p-Fluorophenylalanine, 800 μg/ml; Rapamycin, 200 nM; Tamoxifen, 25 μM; and Tunicamycin, 1 mg/ml). Other growth, sequencing and data processing methods are unchanged. All datasets, excepting Rapamycin, were collected in duplicate and the average fitnesses are presented.

### Hierarchical clustering

Clustering was performed using scipy (version 0.17.0) in Python with the following parameters:

scipy.cluster.hierarchy.linkage(method=‘ward’)

Clustering was performed on 17 vectors representing the fitness effect of each mutant in each condition. In the case of a missing observation for any single mutant, that mutant was excluded from the analysis.

### Principal component analysis

PCA was performed using scikit-learn (version 0.18.1) in Python with the following parameters:

PCA(copy=True, iterated_power='auto', n_components=None, random_state=None,
svd_solver='auto', tol=0.0, whiten=False)

For each compound, the difference in fitness between DMSO and perturbation was calculated; stop codon substitutions were not included. PCA was performed on these 16 vectors. In the case of a missing observation for any single mutant, that mutant was excluded from the analysis.

### ROSETTA ddG predictions

Interface ddG predictions were generated using the Rosetta macromolecular modeling suite, which is freely available for academic use. The git version used was 12e38402d9. For each amino acid position in the MMS/Ubc13 heterodimer, the interface ddG protocol was run as follows: (1) minimize (with constraints to the starting coordinates) the starting wild-type structure (PDB ID: 2GMI). (2) Generate an ensemble of 50 conformational states using Rosetta's backrub application (10,000 trials, temperature 1.2), using residues in an 8 Å radius of the specified amino acid position as backrub pivot residues. (3) Repack, or repack and mutate the side chains of the specified amino acid and the pivot residues from step 2. (4) Minimize (with constraints) the wild-type and mutant structures generated in step 3. (5) For each structure *i* (of 50)*,* we calculate the ddG score as follows:
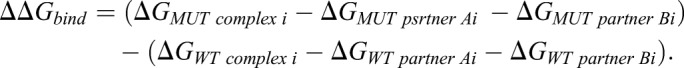
We then average all 50 ΔΔ*G_bind_* scores to obtain the final predicted value.

## Supplementary Material

Supplementary information
