# Supplementary material for: Extending chemical perturbations of the ubiquitin fitness landscape in a classroom setting reveals new constraints on sequence tolerance
Source: Biol Open. 2018 Jul 15;7(7):bio036103. doi: 10.1242/bio.036103 (PMC6078352; doi:10.1242/bio.036103)
Supplement: Supplementary information [file biolopen-7-036103-s1.pdf]

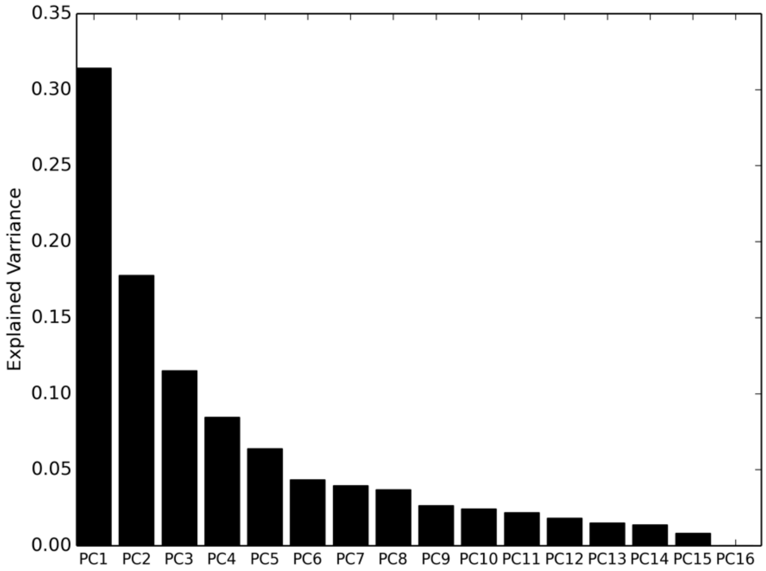

Supplemental Figure 1. Variance explained by the each of first 16 PCs of the difference fitness data.

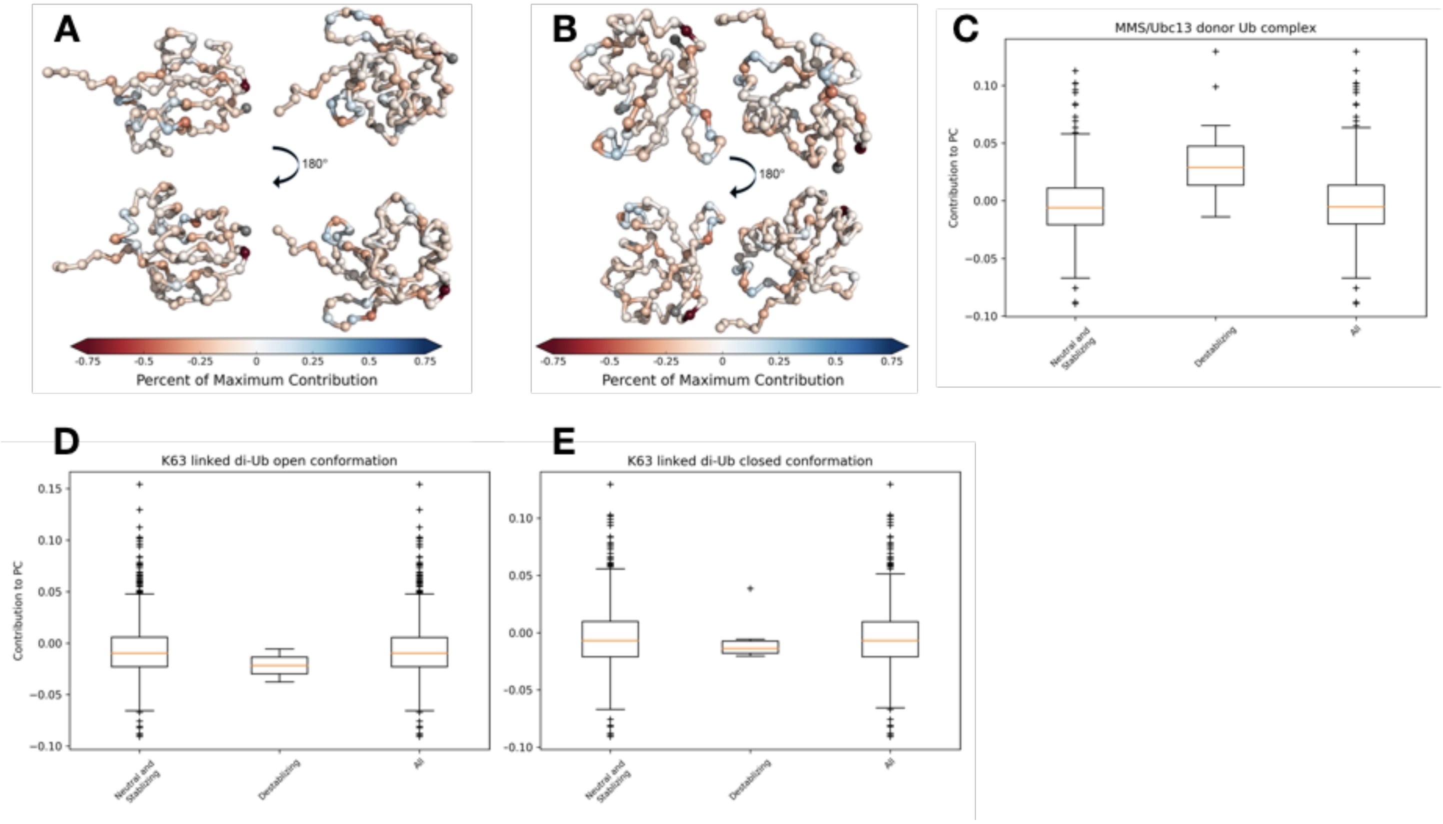

Supplemental Figure 2. Association between PC3 and K63-related structures. **(**A) K63 linked di-Ub open conformation (3H7P) colored by average contribution to PC3. (B) K63 linked di-Ub closed conformation (2N2K) colored by average contribution to PC3. (C) Rosetta calculations reveal a potential association between destabilizing the MMS/Ubc13 donor complex (2GMI) and PC3. Binning mutations as either neutral and stabilizing mutations (Rosetta Energy Units (REU) < 0.75) or destabilizing (REU >= 0.75) reveals a significant difference in PC3 contribution magnitude (Student's t-test, p= 0.000215) for this complex. (D) In contrast, Rosetta calculations reveal no significant difference for the K63 linked di-Ub open conformation (3H7P, Student's t-test, p= 0.887) or (E) for the K63 linked di-Ub closed conformation (2N2K, Student's t-test, p= 0.887)
